# Cellular Virotherapy Increases Tumor-Infiltrating Lymphocytes (TIL) and Decreases their PD-1^+^ Subsets in Mouse Immunocompetent Models

**DOI:** 10.3390/cancers12071920

**Published:** 2020-07-16

**Authors:** Alvaro Morales-Molina, Miguel Ángel Rodríguez-Milla, Alicia Gimenez-Sanchez, Ana Judith Perisé-Barrios, Javier García-Castro

**Affiliations:** 1 Cellular Biotechnology Unit, Instituto de Salud Carlos III, E-28220 Madrid, Spain; alvaromorales102@gmail.com (A.M.-M.); rmilla@isciii.es (M.Á.R.-M.); aliciags89@gmail.com (A.G.-S.); aperibar@uax.es (A.J.P.-B.); 2 Biomedical Research Unit, Universidad Alfonso X el Sabio, E-28691 Madrid, Spain

**Keywords:** oncolytic virus, adenovirus, MSCs, immunotherapy, Celyvir, TILs, T cells, PD-1, renal cancer, melanoma

## Abstract

Oncolytic virotherapy uses viruses designed to selectively replicate in cancer cells. An alternative to intratumoral administration is to use mesenchymal stem cells (MSCs) to transport the oncolytic viruses to the tumor site. Following this strategy, our group has already applied this treatment to children and adults in a human clinical trial and a veterinary trial, with good clinical responses and excellent safety profiles. However, the development of immunocompetent cancer mouse models is still necessary for the study and improvement of oncolytic viroimmunotherapies. Here we have studied the antitumor efficacy, immune response, and mechanism of action of a complete murine version of our cellular virotherapy in mouse models of renal adenocarcinoma and melanoma. We used mouse MSCs infected with the mouse oncolytic adenovirus dlE102 (OAd-MSCs). In both models, treatment with OAd-MSCs significantly reduced tumor volumes by 50% and induced a pro-inflammatory tumor microenvironment. Furthermore, treated mice harboring renal adenocarcinoma and melanoma tumors presented increased infiltration of tumor-associated macrophages (TAMs), natural killer cells, and tumor-infiltrating lymphocytes (TILs). Treated mice also presented lower percentage of TILs expressing programmed cell death protein 1 (PD-1)—the major regulator of T cell exhaustion. In conclusion, treatment with OAd-MSCs significantly reduced tumor volume and induced changes in tumor-infiltrating populations of melanoma and renal cancer.

## 1. Introduction

Oncolytic virotherapy is a form of immunotherapy that uses replication-selective viruses to lyse cancer cells and break immune tolerance in cancer [1,2]. However, their delivery to tumor sites is still challenging, and only a limited fraction of oncolytic viruses manages to reach the target tumor after systemic administration of the therapy, as they are cleared by the immune system [3]. The use of mesenchymal stem cells (MSCs) as cell vehicles for virus delivery applications has been considered, then, as an alternative to intravenous or intratumoral administration of the oncolytic virus alone, as MSCs present tropism for tumors [4,5]. In this way, this cellular virotherapy can be administered in cases in which the tumor is not accessible and may even act in tumors or metastases that have not been found. Furthermore, this “Trojan horse” strategy may also evade the initial immune response of the patient against the viral presence and protect the oncolytic virus when it is transported inside the cell [6]. In fact, different studies suggest that antitumor efficacy of oncolytic adenovirus (OAd) carried by MSCs may even improve the antitumor effect triggered by OAd alone, while treatment with MSCs alone did not induce any antitumor effect [7,8,9].

Following this antitumor strategy, our group has been working for several years on a treatment called Celyvir, consisting of human MSCs loaded with the human OAd ICOVIR-5 [10,11]. This therapy has already been tested in a clinical trial (ClinicalTrials.gov identifier: NCT01844661) of children presenting refractory solid tumors, which resulted in clinical benefits—including two complete remissions—and the remarkable absence of side effects [12,13,14]. A canine version of the treatment using dog MSCs infected with ICOCAV17—a canine OAd homologous to ICOVIR-5—has also been successfully applied in a veterinary trial of dogs presenting spontaneous solid tumors [15,16].

These and other studies have demonstrated that adaptive immune subsets like CD8^+^ T cells are crucial mediators of the induced antitumor immunity in patients treated with oncolytic virotherapies [17,18]. Nevertheless, although some immunocompetent mouse models have been developed [5,19,20], most commonly used preclinical models for studying oncolytic viruses are immunodeficient, which might not represent the real mechanism of action of these viroimmunotherapies, as they present later clearance, higher viral replication, and increased antitumor effects compared to immunocompetent hosts [21]. In addition, most mouse cells are refractory to the replication of human adenovirus, and as a result, the study of human OAd has been almost restricted to xenograft mouse models, which need to be performed in immunodeficient mice. For these reasons, optimum immunocompetent mouse models are necessary for the study and improvement of this and other similar oncolytic viroimmunotherapies.

We here study the antitumor efficacy of a complete murine version of Celyvir, using mouse MSCs loaded with the OAd dlE102 (OAd-MSCs) in immunocompetent mouse models of renal adenocarcinoma and melanoma, including their immune response and mechanism of action. The dlE102 is a modified mouse adenovirus type 1 whose replication is restricted to certain murine cells. In homology to ICOVIR-5, the virus presents a deletion in the high-affinity pRb-binding domain that restricts its replication to cells presenting free E2F1, such as cycling or tumor cells, which are defective in the pRb pathway [22,23].

In both tumor models, treatment with OAd-MSCs significantly reduced tumor volumes by 50% and induced a pro-inflammatory tumor microenvironment. Furthermore, renal adenocarcinoma and melanoma tumors treated with OAd-MSCs presented increased infiltration of innate immune populations, such as tumor-associated macrophages (TAMs) and natural killer (NK) cells, as well as changes in density and activation of tumor-infiltrating lymphocytes (TILs).

## 2. Materials and Methods

### 2.1. Cell Lines

Mouse C57BL/6 MSCs were obtained from abdominal adipose tissue of C57BL/6J mice as previously described [24]. Briefly, adipose tissue was digested with collagenase B (2 mg/mL; Roche, Basel, Switzerland) for 45 min at 37 °C and filtered through a sterile 70 μm nylon mesh cell strainer (Thermo Fisher Scientific, Madrid, Spain). Cells were seeded in a cell culture plate and cultured in complete Dulbecco’s Modified Eagle’s Media (DMEM) (12-604F, Lonza, Barcelona, Spain): DMEM supplemented with 10% FBS (Sigma-Aldrich, St. Louis, MO, USA), streptomycin (100 mg/mL; Lonza), penicillin (100 U/mL; Lonza) and glutamine (2 mM; Lonza) at 37 °C in a humidified atmosphere with 5% CO_2_.

Different mouse tumor cell lines were used: B16-F1 and B16-F10 (melanoma cell lines) and GL261 (glioblastoma cell line) were derived from C57BL/6 mice; the CMT 64-6 clone was derived from a parental CMT 64 cell line [19], a murine, non-small-cell lung carcinoma obtained from C57BL/6J mice; SVZ (glioma cell line) was generated by retroviral expression of EGFR vIII in subventricular zone progenitors from p16/p19 knockout mice, as previously described [25]; Renca (renal cortical adenocarcinoma cell line) was obtained from BALB/c mice; and L929 (connective tissue cell line) was derived from C3H/An mice. All cells were cultured in complete DMEM or RPMI (Roswell Park Memorial Institute), depending on the specific culture conditions, at 37 °C in a humidified atmosphere with 5% CO_2_. All cells were tested for mycoplasma.

### 2.2. Oncolytic Adenovirus

The OAd dlE102 was previously developed and described by Dr. Katherine Spindler’s group [22,23]. For the titration of dlE102, 75,000 cells/well were seeded in a 96-well plate and infected with 50 µL of adenovirus production at different dilutions (1/10–1/10^12^) in triplicate conditions. Thirty-six hours later, an immunofluorescence assay was performed using polyclonal primary antibody anti-adenovirus 5 (ab6982; Abcam, Cambridge, United Kingdom) at 1/800, followed by a secondary anti-rabbit antibody (Invitrogen, Carlsbad, CA, USA) at 1/1000. Positive cells were counted at 1/10^4^ and 1/10^5^ triplicate dilutions using a fluorescence microscope. The mean of the six wells was used to calculate the infectious units per mL for OAd dlE102.

Infections of cells with the OAd dlE102 were performed at multiplicity of infection (MOI) of 1, 20, and 200 during 1 h at 37 °C in DMEM without FBS. Cells were washed two times with PBS to remove the virus from the cell culture supernatant. Viral production from infected MSC or tumor cells was assessed by collection of the supernatant 72 h after infection. Virus particles in the culture supernatants were quantified using L929 cells at the same ratio and conditions as described above. An immunofluorescence staining was also performed using the previously described protocol.

### 2.3. Western Blot and NF-κB Activity

Total proteins were extracted with sodium dodecyl sulfate (SDS) sample buffer and 1:100 protease inhibitor cocktail (Sigma-Aldrich) 3 and 24 h after infection. Proteins were separated by electrophoresis, transferred to polyvinylidene difluoride (PVDF) membranes (Bio-Rad Laboratories, Madrid, Spain), and blocked with 2% milk in tris-buffered saline. Mouse monoclonal phospho-c-Jun (clone KM-1; Santa Cruz Biotechnology, TX, USA), β-actin (clone AC-15; Sigma-Aldrich), rabbit monoclonal phospho-Akt (Ser473, clone 2118-1; Abcam), and phospho-Stat1 (Ser727; Cell Signaling Technology, Leiden, The Netherlands) were used as primary antibodies. Polyclonal goat anti-rabbit and anti-mouse immunoglobulins/HRP (Agilent Dako, Santa Clara, CA, USA) were used as secondary antibodies for 1 h at RT. The HRP signal was detected with Immobilon Western Chemiluminiscent HRP Substrate (Merck Millipore, Madrid, Spain).

The activation of nuclear factor kappa B (NF-κB) was evaluated by using a luciferase reporter system [26]. MSCs were transduced overnight with a non-replicative lentiviral vector that contains the pHAGE NF-κB-TA-LUC-UBC-GFP-W plasmid (Addgene, Watertown, MA, USA; plasmid #49343; http://n2t.net/addgene:49343). Twenty-four hours after infection with dlE102 at MOIs 1 and 10, cell lysis for total protein extraction was carried out, and luciferase activity was assayed with the Luciferase Assay System (Promega Corporation, Madrid, Spain).

### 2.4. Animal Models

For in vivo tracking of OAd-MSCs to the Renca tumors, MSCs were labeled with 8.33 mg/mL DiR buffer (DiIC18(7) or 1,1′-dioctadecyltetramethyl indotricarbocyanine Iodide) for 30 min at 37 °C, according to the manufacturer (Caliper Lifesciences, Waltham, MA, USA), prior to infection with dlE102 (MOI 1). DiR labeling was assessed by flow cytometry. Then, 2 × 10^6^ DiR-labeled OAd-MSCs per mouse were intraperitoneally injected in BALB/c mice bearing subcutaneous Renca tumors. Non-labeled OAd-MSCs were used as a negative control. Seventy-two hours later, tumors were obtained and digested with collagenase IV (1 mg/mL). The DiR signal was measured from the tumor cell suspension by flow cytometry. Detection of dlE102 in the tumor was determined as previously described [27], using quantitative PCR (qPCR) primer sets specific for mouse adenovirus type 1 [28]: forward, 5′-GGCCAACACTACCGACACTT-3′; reverse, 5′-TTTTGTCCTGTGGCATTTGA-3′. Copies of dlE102 were adjusted to the basal expression of GAPDH (forward, 5′-AGGTCGGTGTGAACGGATTTG-3′; reverse, 5′-GGGGTCGTTGATGGCAACA-3′) and normalized to one gram of tumor tissue.

For in vivo antitumor experiment, 2.5 × 10^6^ Renca cells and 1 × 10^6^ B16-F1 tumor cells were implanted subcutaneously in 7-week-old BALB/c and C57BL/6 mice, respectively. When the tumors were measurable (day 21 and day 5, respectively), the first dose of PBS, MSCs (1 × 10^5^ cells/mouse) or OAd-MSCs (1 × 10^5^ cells/mouse at MOI 1) was inoculated intraperitoneally. A total of three doses separated by 4–5 days were administered. As an additional control group, a single dose of OAd dlE102 (1 × 10^6^ viral particles/mouse) was intratumorally administered in mice bearing Renca tumors. Tumor length (L), width (W), and height (H) were measured with a caliper periodically, and tumor volume was calculated as (L × W × H) × π/6. Thirteen days after the first treatment, mice were sacrificed, and tumors were processed for flow cytometry. The experiment was repeated two times (*n* = 8*–*14 mice per group). Sections of the tumor samples were directly frozen, while others were fixed and embedded within Tissue-Tek. Eight μm-thick sections were stained with hematoxylin and eosin following standard protocol. Representative maps of the tumors were obtained using the scanner OPTISCAN10 (OPTIKA), and detailed images were acquired on a ZEISS Primo Star microscope using a 10× objective and an Axiocam ERc 5 s camera (ZEISS, Madrid, Spain).

All procedures involving animals were approved on 2 December 2015, by the Animal Research and Welfare Ethics Committee (Comité de Ética de la Investigación y de Bienestar Animal) of Instituto de Salud Carlos III (PROEX 347/15), where the studies were conducted.

### 2.5. Flow Cytometry

Extracted tumors were digested with collagenase IV (1 mg/mL) in agitation for 40 min at 37 °C and mechanically homogenized using a Potter–Elvehjem PTFE pestle when necessary. Cell suspensions were filtered through a sterile 70 μM nylon mesh cell strainer, and red blood cells were lysed by incubation with Quicklysis buffer (Cytognos, Salamanca, Spain). Cell suspensions were blocked with mouse FcR Blocking (Miltenyi Biotec, Madrid, Spain) for 15 min and incubated with the following mouse monoclonal antibodies for 20 min at 4 °C: CD45 (clone 30-F11), CD3 (clone 145-2C11), CD4 (clone GK1.5), CD8 (clone 53-6.7), CD11b (clone M1/70), CD11c (clone N418), CD206 (clone C068C2), MHCII (clone M5/114.15.2), Ly6C (clone AL-21), Ly6G (clone 1A8-Ly6g), CD49b (clone DX5). All these antibodies were obtained from eBioScience-Thermo Fisher Scientific, while the programmed cell death protein 1 (PD-1) (clone 29F.1A12) came from BioLegend (San Diego, CA, USA). After incubation, cells were washed and labeled with the viability marker 7-aminoactinomycin (7AAD; Thermo Fisher Scientific) for 10 min at RT. Samples were acquired with MACSQuant Analyzer cytometer and analyzed using MACSQuantify 2.13 software (Miltenyi Biotec). Density of the following immune cell populations was normalized to tumor volume to allow for comparisons: leukocytes (CD45^+^); T cells (CD45^+^ CD3^+^), subclassified in helper T cells (CD4^+^), and cytotoxic T cells (CD8^+^); and NK cells (CD45^+^ CD11c^+^ CD49b^+^); myeloid cells (CD45^+^ CD11b^+^), subclassified into monocytes (Ly6G^−^ MHCII^−^), macrophages (Ly6G^−^ MHCII^+^ CD11c^low^), dendritic cells (Ly6G^−^ MHCII^+^ CD11c^high^), and neutrophils (Ly6G^+^ MHCII^−^). M2, DC2, and N2 subsets were also considered (CD206^+^).

### 2.6. Cytokine Array

The frozen tumors were homogenized, and the total proteins were extracted as described above. Functional markers of tumors treated with PBS or OAd-MSCs were analyzed by cytokine array assay following manufacturer’s indications (Proteome Profiler Mouse Array Panel A kit, ARY006; R&D Systems, Minneapolis, MN, USA). Cytokine expression was measured semi-quantitatively by pixel density of duplicated spots using ImageJ software. Differentially expressed proteins were further studied with STRING software to analyze the biological process in which they are involved.

### 2.7. Statistical Analysis

Data was analyzed and graphed with GraphPad Prism 8 (GraphPad Software, San Diego, CA, USA). In vitro results were expressed as the mean + SD, and in vivo results were expressed as the mean + SEM (standard error of the mean), as indicated in the figure legends. Significant differences were determined using unpaired parametric or non-parametric tests (*t*-test or Mann–Whitney *U* test, respectively): * *p* ≤ 0.05, ** *p* ≤ 0.01, *** *p* ≤ 0.001.

## 3. Results

### 3.1. Mouse Oncolytic Adenovirus dlE102 Replicates in Mouse MSCs, and Does Not Activate Pro-Inflammatory Pathways

**Figure 1:**
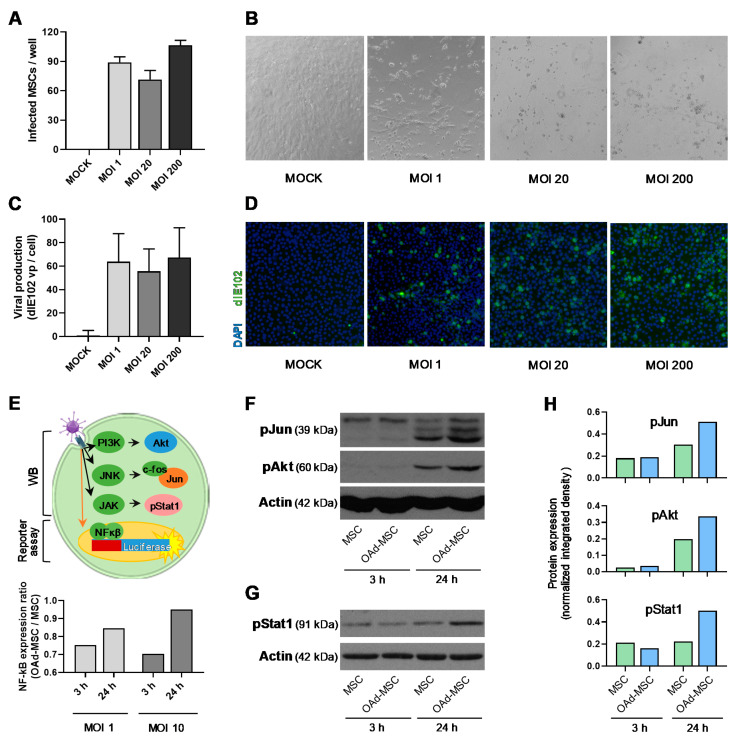
Viral production and signaling of mouse mesenchymal stem cells (MSCs) infected with dlE102. (**A**) Infected C57BL/6 MSCs per 96 wells, 48 h after infection with oncolytic adenovirus (OAd) dlE102 at multiplicities of infection (MOIs) 1, 20, and 200. Non-infected MSCs were used as a negative control (MOCK). (**B**) Representative images (20×) showing cytopathic effect in MSCs after infection with OAd dlE102 at MOIs 1, 20, and 200. (**C**) Viral production of MSCs infected with OAd dlE102 at MOIs 1, 20, and 200 (vp = viral particles). (**D**) Representative images (20×) showing adenoviral staining (green) in L929 cells cultured for 48 h with supernatants from infected MSCs. (**E**) Scheme showing the studied molecular signaling after adenoviral infection. Activation of Jun, Akt, and phospho-Stat1 (pStat1) is detected by Western blot (WB), while activation of the NF-κB pathway is studied using a luciferase reporter system. Graph represents the NF-κB activation at 3 and 24 h expressed as an OAd-MSC/MSC ratio. (**F**,**G**) Protein expression of phospho-Jun (pJun), phospho-Akt (pAkt), pStat1, and Actin analyzed by WB at 3 and 24 h after infection with dlE102. Mock-infected MSCs were used as a control. Complete WB images can be found in Figures S3–S7. (**H**) Quantification of protein expression of pJun, pAkt, and pStat1 expressed as integrated density normalized to loading controls.

As part of their role as cell carriers in our cellular virotherapy, MSCs must be able to support the replication and production of new viral particles after infection with the oncolytic virus, so we studied the replication of mouse OAd dlE102 in C57BL/6 MSCs at different MOIs. Prior to this, we first assessed the viral infection of the MSCs and the triggered cytopathic effect, which presented similar results using MOIs 1, 20, or 200 (Figure 1A,B). In the same way, viral replication was successfully addressed, presenting similar outcomes in those MSCs infected at MOIs 1, 20, or 200 (Figure 1C,D). Furthermore, these new virus particles produced by MSCs were able to successfully infect L929 cells (Figure 1D), which confirmed their capacity of infection.

As inflammatory profiles of MSCs after adenoviral infection seems to play a relevant role in the antitumor effect of the cellular virotherapy [13], we studied the molecular signaling of infected MSCs at 3 and 24 h (Figure 1E). NF-κB activation, which is considered the main initiating cellular event in response to infection by pathogens [29], was studied using a luciferase reporter system [26]. However, no relevant activation in the expression of luciferase was observed at MOIs 1 or 10 at any time (Figure 1E). Previous studies have demonstrated a higher expression of phospho-Akt (pAkt), and phospho-Jun (pJun) in C57BL/6 MSCs after infection with the human adenovirus ICOVIR-5 [19,27], so we also studied the activation of these pathways after infection with dlE102. Interestingly, expression of pJun and pAkt in infected MSCs (OAd-MSCs) was only slightly increased or similar to the observed in control MSCs at 3 and 24 h (Figure 1F and Figures S3–S5). The study of JAK/STAT signaling pathway through expression of phospho-Stat1 (pStat1) showed similar outcomes (Figure 1G,H and Figures S6 and S7). These results indicate that the infection of mouse MSCs with mouse OAd dlE102 does not induce high activation of the typical pro-inflammatory pathways.

### 3.2. Mouse Oncolytic Adenovirus dlE102 Successfully Replicates in Mouse Tumor Cells

**Figure 2:**
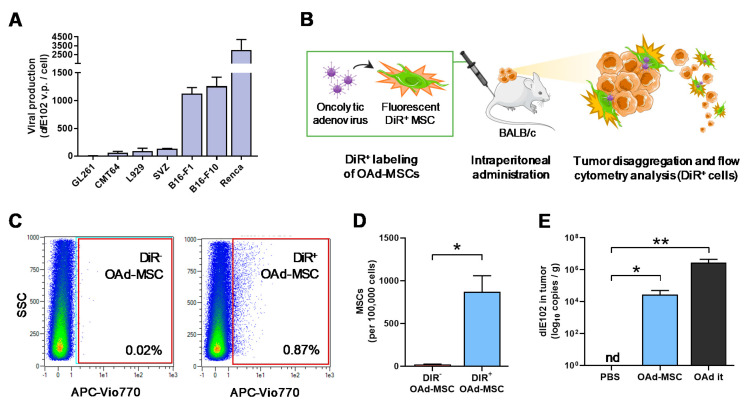
In vitro viral production in mouse tumor cell lines and in vivo tumor-homing of OAd-MSCs. (**A**) Viral production in different mouse tumor cell lines after OAd dlE102 infection (*n* = 3). (**B**) Scheme showing the experimental design. MSCs are labeled with the fluorescent marker DiR and infected with dlE102 prior to intraperitoneal administration into tumor-bearing BALB/c mice. Seventy-two hours later, tumors are harvested, processed, and analyzed. (**C**) Analysis by flow cytometry of dlE102-MSCs in the tumors (DiR^+^: DiR-labeled OAd-MSCs; DiR^−^: non-labeled OAd-MSCs as a negative control). (**D**) Quantification of migrated MSCs infected with OAd (*n* = 4). (**E**) Detection by qPCR of OAd dlE102 in Renca tumors treated with PBS (negative control), OAd-MSCs, or intratumorally administered OAd (OAd it, positive control), expressed as dlE102 copies per gram of tumor. nd: not detectable; unpaired *t*-test, * *p* < 0.05, ** *p* < 0.01.

To study the in vitro antitumor efficacy, viral production after infection with OAd dlE102 was studied in a panel of different mouse tumor cell lines. Cancer cells were then infected at MOI 10, and the resultant supernatants after 72 h were added to the L929 cell line to assess viral production. From the studied panel, Renca cells followed by B16-F1 and B16-F10 melanoma cells presented the highest viral production compared to the other murine tumor cell lines (Figure 2A). These results demonstrate that OAd dlE102 can replicate in different mouse tumor cell lines.

### 3.3. MSCs and Oncolytic Adenovirus dlE102 Are Detected in the Tumor Site after Systemic Administration of OAd-MSCs

To assess the homing of OAd-MSCs to the tumor site, MSCs were labeled with the fluorescent marker DiR and later infected with OAd dlE102, obtaining fluorescent DiR^+^ OAd-MSCs. The treatment was administered in Renca tumor-bearing BALB/c mice; tumors were obtained 72 h later and analyzed for DiR^+^ signal by flow cytometry (Figure 2B). The presence of migrated MSCs (DiR^+^ events) was observed in tumors from mice treated with fluorescent OAd-MSCs (0.87% of all tumor disaggregation), while, as expected, no DiR^+^ events were detected in control mice treated with DiR^-^ OAd-MSCs (Figure 2C,D). OAd dlE102 presence in the tumors was also confirmed by qPCR in mice treated with OAd-MSCs (Figure 2E). These results demonstrate that MSCs successfully transport and deliver the OAd to the tumor site after systemic administration of OAd-MSCs.

### 3.4. Treatment with OAd-MSCs Presents Significant Antitumor Efficacy and Induces a Pro-Inflammatory Profile in a Renal Cancer Model

**Figure 3:**
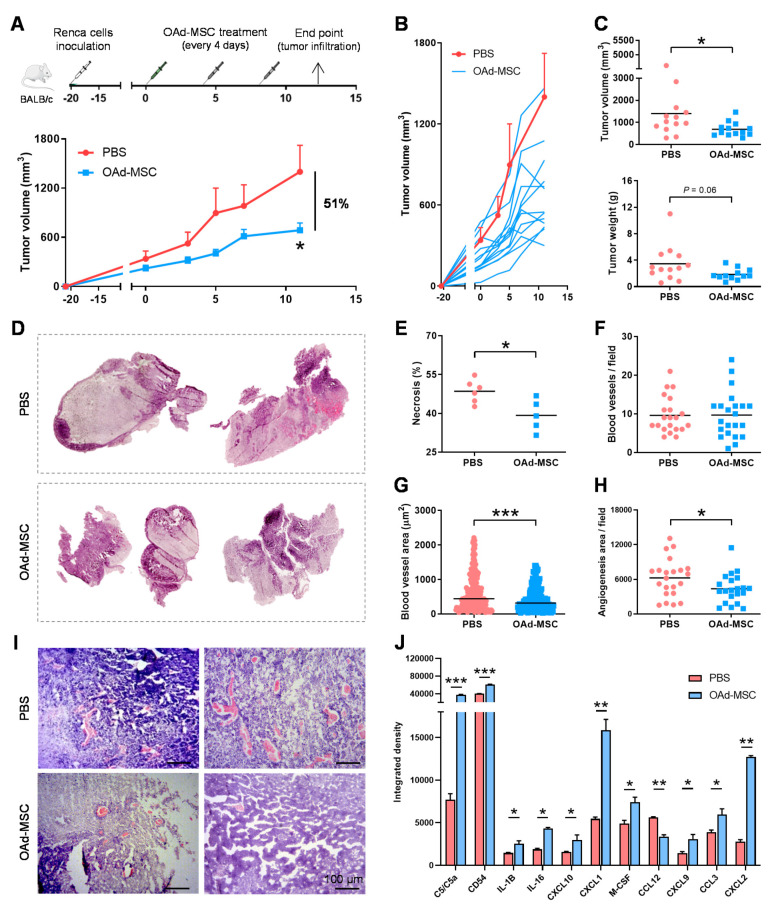
In vivo antitumor efficacy of OAd-MSCs in a mouse model of renal adenocarcinoma. (**A**) Graph represents the experimental design and antitumor effect of OAd-MSCs in BALB/c mice bearing subcutaneous Renca tumors. Lines represent the mean + standard error of the mean (SEM) of the tumor volume in groups treated with PBS (red; *n* = 13) and OAd-MSCs (blue; *n* = 12) (Mann–Whitney *U* test). (**B**) Follow-up of tumor volume in mice treated with OAd-MSCs (individual values, blue lines) and the control group (mean + SEM, red line). (**C**) Tumor volume and weight of groups treated with PBS and OAd-MSC at end point (Mann–Whitney *U* test). (**D**,**E**) Necrosis was quantified in hematoxylin- and eosin-stained sections from control and treated tumors (*n* = 5–6). (**F**–**H**) Angiogenesis was studied by (**F**) quantification of number of tumor blood vessels per field, (**G**) area per blood vessel, and (**H**) total angiogenesis area per field (µm^2^) (Mann–Whitney *U* test). (**I**) Representative images of hematoxylin- and eosin-stained sections from control and treated tumors. (**J**) Quantification by integrated density of pro-inflammatory cytokines differentially expressed in tumors treated with PBS (red) or OAd-MSC (blue) (unpaired *t*-test). * *p* < 0.05, ** *p* < 0.01, *** *p* < 0.001.

For the establishment of an in vivo immunocompetent model of renal cancer, a first antitumor efficacy experiment treating mice with MSCs alone, OAd-MSCs, or intratumoral OAd dlE102 was performed. Interestingly, tumors from mice treated with intraperitoneal OAd-MSCs or intratumoral OAd presented similar antitumor effect, while those treated with non-infected MSCs were similar to the PBS control group (Figure S1A,B). With this accomplished, in vivo experiments were performed to study the antitumor efficacy and mechanism of action of OAd-MSCs in this renal cancer model (Figure 3A). At the end point, tumor volumes of mice treated with OAd-MSCs were significantly smaller (51%) than those from the PBS control group (Figure 3A,B). The same tendency was also observed in tumor weight at the end point (Figure 3C). Histological analysis showed that tumors treated with OAd-MSCs presented significant lower percentages of necrotic tissue than those from PBS control group (Figure 3D,E). Angiogenesis was also studied by analysis of tumor blood vessels. Interestingly, although the number of blood vessels was similar in tumors treated with PBS or OAd-MSCs (Figure 3F), those from the OAd-MSC group presented significant smaller sizes (Figure 3G). As a result, total angiogenesis area was significantly lower in tumors treated with OAd-MSCs than in those treated with PBS (Figure 3H). Analysis of pro-inflammatory cytokines showed significant higher expression of C5/C5a, CD54, IL-1β, IL-16, CXCL10, CXCL1, M-CSF, CXCL9, CCL3, and CXCL2 (10 out of 40 cytokines) in tumors treated with OAd-MSCs compared to the control tumors, while only CCL12 was reduced (Figure 3H and Figure S2A). These results indicate that treatment with OAd-MSCs induces antitumor effect in vivo, reduces necrosis and angiogenesis, and increases the pro-inflammatory profile in renal cancer.

### 3.5. Treatment with OAd-MSCs Increases Tumor-Infiltrating Immune Cells in a Renal Cancer Model

**Figure 4:**
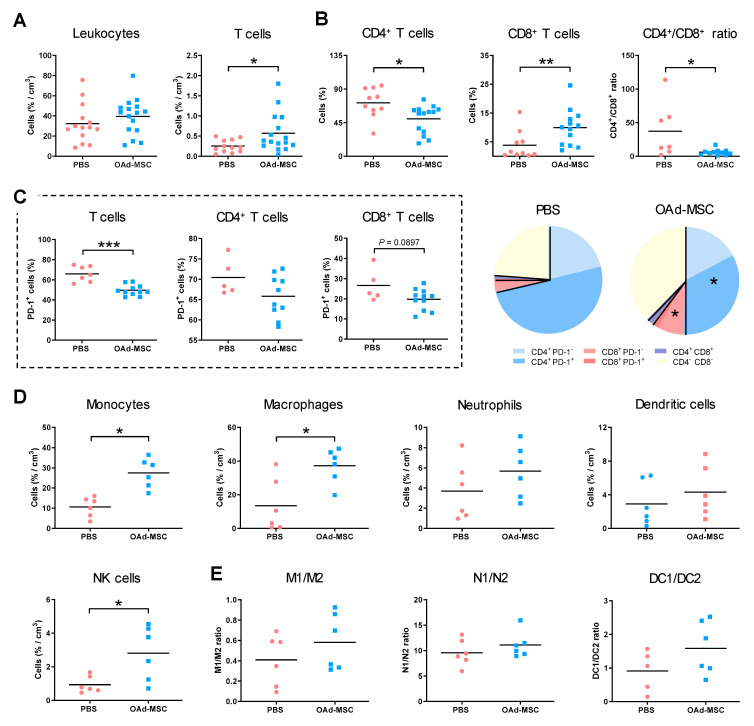
Tumor-infiltrating immune cells in renal adenocarcinoma. (**A**) Density of tumor-infiltrating lymphocytes (TILs) and leukocytes at end points, expressed as percentage per cm^3^ of tumor. (**B**) Percentage of CD4^+^ and CD8^+^ T cell subsets from T cells, as well as CD4^+^/CD8^+^ ratio (*n* = 10–15). (**C**) Percentage of TIL, CD4^+^, and CD8^+^ T cells expressing programmed cell death protein 1 (PD-1) (*n* = 5–11). The pie chart shows percentages of CD4^+^ PD-1^−^ (blue), CD4^+^ PD-1^+^ (dark blue), CD8^+^ PD-1^−^ (red), CD8^+^ PD-1^+^ (dark red), CD4^+^ CD8^+^ (purple), and CD4^−^ CD8^−^ (yellow) T cell subsets from the TILs. (**D**) Density of tumor-infiltrating innate immune cells and natural killer (NK) cells (*n* = 6). (**E**) Ratio of pro-inflammatory (1) and anti-inflammatory (2) status of macrophages, neutrophils, and dendritic cells (*n* = 5–6). (Mann–Whitney *U* test) * *p* < 0.05, ** *p* < 0.01, *** *p* < 0.001.

Flow cytometry analysis of intratumoral infiltration of immune subpopulations showed a similar density of leukocytes in tumors from the PBS or OAd-MSC groups. However, a significant increase in tumor-infiltrating lymphocytes (TILs) was observed in tumors treated with OAd-MSCs (Figure 4A), as well as a significant reduction of the CD4^+^/CD8^+^ ratio, due to the reduced and increased levels of CD4^+^ and CD8^+^ T cells, respectively (Figure 4B). Apart from these changes in ratios, analysis of T cell exhaustion showed a significantly lower percentage of TILs expressing programmed cell death protein 1 (PD-1) in OAd-MSCs than in PBS-treated tumors, which was similarly observed in CD4^+^ and CD8^+^ subsets (Figure 4C). Furthermore, analysis of innate immune cells showed a significant higher density of NK cells, TAMs, and monocytes in mice treated with OAd-MSCs (Figure 4D). No significant differences were found in their anti-inflammatory/pro-inflammatory ratios (Figure 4E). These results indicate that the treatment of renal cancer with OAd-MSCs induces an increase of tumor infiltration of innate and adaptive immune cells, as well as a reduction of the exhaustion status of TILs.

### 3.6. Treatment with OAd-MSCs Presents Significant Antitumor Efficacy and Induces a Pro-Inflammatory Profile in a Melanoma Model

**Figure 5:**
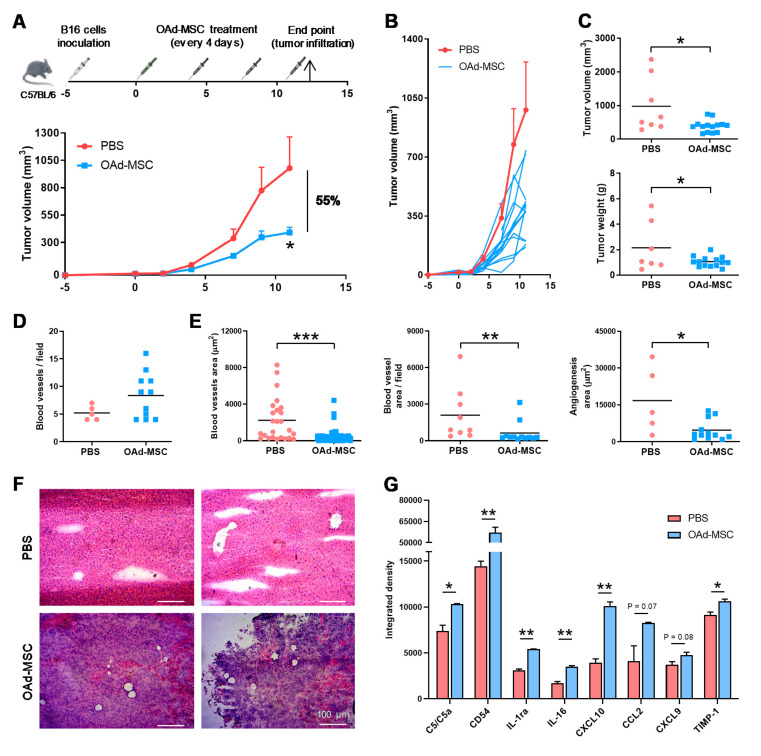
In vivo antitumor efficacy of OAd-MSCs in a mouse model of melanoma. (**A**) Graph represents the experimental design and antitumor effect of OAd-MSCs in C57BL/6 mice bearing subcutaneous B16 tumors. Lines represent the mean + SEM of the tumor volume in groups treated with PBS (red; *n* = 8) and OAd-MSC (blue; *n* = 14) (Mann–Whitney *U* test). (**B**) Follow-up of tumor volume in mice treated with OAd-MSCs (individual values, blue lines) and control group (mean + SEM, red line). (**C**) Tumor volume and weight of groups treated with PBS and OAd-MSCs at the end points (Mann–Whitney *U* test and unpaired *t*-test, respectively). (**D**,**E**) Angiogenesis was studied by (**D**) quantification of the number of tumor blood vessels per field, (**E**) area per blood vessel, blood vessel area per field, and total angiogenesis area (μm^2^) (Mann–Whitney *U* test). (**F**) Representative images of hematoxylin and eosin-stained sections from control and treated tumors. (**G**) Quantification by integrated density of pro-inflammatory cytokines differentially expressed in the tumors treated with PBS (red) and OAd-MSCs (blue) (unpaired *t*-test). * *p* ≤ 0.05, ** *p* < 0.01, *** *p* < 0.001.

The antitumor efficacy of treatment with OAd-MSCs was also studied in an in vivo, immunocompetent model of B16 melanoma cells (Figure 5A). At the end point, mice treated with OAd-MSCs presented significant smaller tumor volumes (56%) and tumor weights than those treated with PBS (Figure 5A–C). Although a similar number of blood vessels was observed in tumors treated with either PBS or OAd-MSCs (Figure 5D), the size of blood vessels from the OAd-MSC group was significantly smaller, which resulted in significant reduced total areas of angiogenesis (Figure 5E,F). Analysis of pro-inflammatory cytokines showed significantly higher expression of C5/C5a, CD54, IL-1ra, IL-16, CXCL10, CCL2, CXCL9, and TIMP-1 (8 out of 40 cytokines) in tumors treated with OAd-MSCs than in control tumors (Figure 5G). These results indicate that treatment with OAd-MSCs significantly reduces tumor volume and angiogenesis in melanoma, while increasing its pro-inflammatory profile, similar to the effect observed in renal cancer.

### 3.7. Treatment with OAd-MSCs Induces Changes in Tumor-Infiltrating Immune Cells in a Melanoma Model

**Figure 6:**
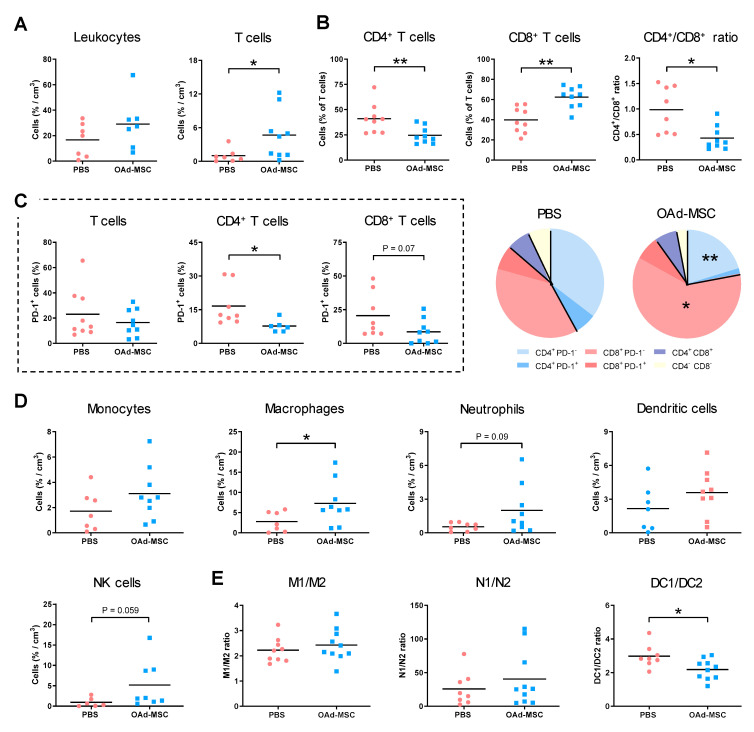
Tumor-infiltrating immune cells in melanoma. (**A**) Density of tumor-infiltrating lymphocytes (TILs) and leukocytes at the end point, expressed as percentage per cm^3^ of tumor. (**B**) Percentage of CD4^+^ and CD8^+^ T cell subsets from T cells, as well as the CD4^+^/CD8^+^ ratio. (**C**) Percentage of TILs, CD4^+^, and CD8^+^ T cells expressing PD-1. Pie chart shows percentages of CD4^+^ PD-1^−^ (blue), CD4^+^ PD-1^+^ (dark blue), CD8^+^ PD-1^−^ (red), CD8^+^ PD-1^+^ (dark red), CD4^+^ CD8^+^ (purple), and CD4^−^ CD8^−^ (yellow) T cell subsets from TILs. (**D**) Density of tumor-infiltrating innate immune cells and NK cells; (**E**) Ratio of pro-inflammatory (1) and anti-inflammatory (2) status of macrophages, neutrophils, and dendritic cells (*n* = 7–9). (Mann-Whitney *U* test): * *p* < 0.05, ** *p* < 0.01.

Flow cytometry analysis of intratumoral infiltration showed that the density of leukocytes was similar in tumors treated with PBS or OAd-MSCs, but the percentage of TILs was significantly increased in the OAd-MSC group (Figure 6A). This significant increase was also observed in the CD8^+^ T cell subset of tumors treated with OAd-MSCs, while the CD4^+^ T cell subset was decreased. As a result, a significant reduction of the CD4^+^/CD8^+^ ratio was observed in tumors treated with OAd-MSCs (Figure 6B). Furthermore, a significantly lower percentage of TILs expressing PD-1 was observed in the OAd-MSC group, which was also observed in the CD4^+^ and CD8^+^ T cell subsets (Figure 6C). Furthermore, analysis of innate immune cells showed higher density of NK cells and TAMs in tumors treated with OAd-MSCs (Figure 6D), along with a significant decrease in the anti-inflammatory/pro-inflammatory dendritic cell (DC1/DC2) ratio (Figure 6E). These results indicate that treatment of melanoma with OAd-MSCs also induces an increased tumor infiltration of innate and adaptive immune cells, as well as a reduction of the exhaustion status of TILs.

## 4. Discussion

The use of MSCs loaded with an oncolytic virus constitutes a promising antitumor treatment that is being currently studied in clinical trials of prostate cancer (ClinicalTrials.gov identifier: NCT01983709), ovarian cancer (ClinicalTrials.gov identifier: NCT02530047 and NCT02068794), head and neck cancer (ClinicalTrials.gov identifier: NCT02079324), lung cancer (ClinicalTrials.gov identifier: NCT03298763), and high-grade gliomas (ClinicalTrials.gov identifier: NCT03896568). In this regard, our group has been working for several years on a treatment called Celyvir, consisting of human MSCs infected with the human OAd ICOVIR-5 [12,13,14]. However, for an appropriate study of these cellular virotherapies, their mechanisms of action and immune response, development of more realistic animal models are still necessary. Here, we have developed and studied the antitumor effects of a complete murine version of Celyvir in immunocompetent mouse models for renal adenocarcinoma and melanoma.

As mouse cells do not support the complete replication cycle of human adenovirus, we firstly assessed viral infection and replication of mouse OAd dlE102 in mouse MSCs and different mouse tumor cells, which was also recently confirmed by Franco-Luzón et al. [30]. Considering that the results were similar to those observed using human MSCs infected with the human OAd, this mouse system may mimic more efficiently the viral mechanism occurring in human cellular viroimmunotherapies.

Although adenoviral infection typically induces the activation of inflammatory or cytotoxic responses from the host cell against the virus [31], NF-κB pathway activation was not induced when mouse MSCs were infected with the OAd dlE102. This is in line with the results obtained by Molloy et al. [32] after in vivo infection of mouse adipose tissue with mouse adenovirus type 1, in which no significant differences in pro-inflammatory mediators were observed between infected and control mice. This opposite response to the species-specific adenoviral infection by human and mouse cells may be explained by a different molecular evolution of each adenovirus species with respect to its host [33,34]. In addition, as human MSCs presenting lower pro-inflammatory profiles resulted in better clinical outcome in patients from clinical trials [13], we consider that the innate, low pro-inflammatory response of mouse MSCs after infection with dlE102 may also enhance the antitumor effect of the therapy. This remains an interesting approach to be studied for the improvement of Celyvir.

Our study shows a comparative analysis of the antitumor effects of mouse MSCs infected with the OAd dlE102 for the treatment of renal cancer and melanoma. In both cases, tumor volumes of the treated mice were significantly reduced, by more than 50%. Interestingly, it has been recently published that treatment with OAd-MSCs in a mouse model of neuroblastoma also showed an antitumor efficacy of 50% [30], which may suggest a similar mechanism of action for this therapy independent from the tumor type.

This obtained antitumor effect remarkably improves the results observed in previous immunocompetent mouse models using mouse MSCs and the human OAd ICOVIR-5, in which tumor growth was only reduced by 35% [19,20]. Similarly, while no differences in tumor angiogenesis were observed in the previous models [19], a significantly reduced angiogenesis area was observed in renal cancer and melanoma tumors treated with our mouse version of Celyvir, which correlates with better clinical outcome in a wide variety of cancers [35,36]. Furthermore, tumor necrosis was also reduced in renal tumors treated with OAd-MSCs, consistent with a meta-analysis of renal cell carcinoma, which demonstrates that tumor necrosis is associated with poor overall survival [37]. As decreased tumor angiogenesis and necrosis usually correlate to better prognosis, it would be of interest to study this parameter in human and canine patients treated with cellular viroimmunotherapies in clinical trials.

Besides the tumor type, the studied mouse strains generally show a preferential immune response: BALB/c mice is a prototypical Th2-type mouse strain, while C57BL/6 mice is a prototypical Th1 immune response [38,39]. Despite this fact, the treatment induced similar changes in the number and activation of innate and adaptive immune cells in both renal cancer and melanoma models.

Analysis of tumor proteins showed that treatment with OAd-MSCs induced an inflammatory effect in melanoma and renal adenocarcinoma tumors. The highest expression of CD54 (also known as ICAM-1) in both models has been previously associated with a lower relapse of melanoma tumors after adoptive T cell therapy [40] and with an antitumor effect in Renca tumors [41,42]. Interestingly, the STRING analysis of the five cytokines that are significantly increased in both models after treatment with OAd-MSCs points to T cell migration as the main biological process in which they are involved (Figure S2C); this may explain the increased infiltration of TILs observed in treated tumors. As demonstrated by Moreno et al. [9], the higher density of TAMs and NK cells in tumors treated with OAd-MSCs may also play a determinant role in the establishment of the antitumor, pro-inflammatory environment, as well as the subsequent adaptive immune response, as the NK cell-mediated killing of virus-infected cells also impacts on T cell responses [43].

Results from previous preclinical models and veterinary and human clinical trials using this therapy have also shown an increase or activation of TILs in the tumor biopsies of treated patients [12,16,19,20], as observed in our results. This increase of TILs and the CD8^+^ T cell subset in the tumor microenvironment is largely associated with a favorable prognosis and survival in melanoma, renal cancer, and other solid tumors [17,18]. Thus, this complete mouse version of cellular virotherapies may represent more efficiently the immune response occurring in real patients from clinical trials.

T cell exhaustion is a common state of T cells in the tumor microenvironment, which leads to cancer immune evasion [44]. The PD-1 receptor, expressed on the surface of T lymphocytes, binds to the PD-1 ligands of tumor cells and represses the T cell activation signal. As PD-1 is the major regulator of T cell exhaustion, together with other inhibitory receptors like CTLA-4 or TIM-3, T cells with high PD-1 expression lose the ability to eliminate cancer, and PD-1^+^ TILs are therefore associated with poor clinical outcome [45,46,47]. Interestingly, tumors treated with OAd-MSCs presented a lower percentage of TILs expressing PD-1 than those from the control group. More specifically, treated tumors presented lower expression of PD1^+^ in both the CD4^+^ and CD8^+^ T cell subsets, which has been associated with better five-year overall survival rates in patients with gastric cancer [48].

These changes in the tumor microenvironment might be induced by the adenoviral infection or the oncolytic and pro-inflammatory effects of the therapy. Regarding the first, it has been published that infection with serotype adenoviral vectors alternative to adenoviral 5 vectors—as dlE102 should be considered—did also induce memory T cells with enhanced functionality and reduced PD-1 expression [49,50]. More interestingly, a recent publication has also demonstrated a reduction in PD-1^hi^ Tim-3^+^ CD8^+^ T cells after infection with an oncolytic virus [51], thus reducing the T cell exhaustion in a similar way as our results. Thus, we hypothesize that treatment with OAd-MSCs may reverse T cell exhaustion, due to the triggered pro-inflammatory changes in the tumor microenvironment derived from the oncolytic virus. Overall, the reported results reinforce the idea that the antitumor efficacy of oncolytic virotherapy is not only derived from the lytic cycle of the virus, but also from the capacity of the treatment to activate the immune response [52], which accentuates the importance of using immunocompetent mouse models for the study of the therapy.

## 5. Conclusions

In conclusion, treatment with OAd-MSCs significantly reduced tumor volume and induced changes in innate and adaptive tumor-infiltrating populations of melanoma and renal cancer, including the exhaustion status of TILs. The results obtained in these mouse models may help to deeply understand the mechanism of action and improve the efficacy of Celyvir and other cellular oncolytic virotherapies.

## Supplementary Materials

The following are available online at https://www.mdpi.com/2072-6694/12/7/1920/s1, Figure S1: In vivo antitumor efficacy of dlE102 and OAd-MSCs in a mouse model of renal adenocarcinoma, Figure S2: Pro-inflammatory profile in control and treated Renca and B16 tumors, Figure S3: Complete WB of phospho-Jun (blot on the left), corresponding to Figure 1F, Figure S4: Complete WB of phospho-Akt (blot on the left), corresponding to Figure 1F, Figure S5: Complete WB of Actin, corresponding to Figure 1F, Figure S6: Complete WB of pospho-Stat-1 Ser727 (blot on the left), corresponding to Figure 1G, Figure S7: Complete WB of Actin, corresponding to Figure 1G.
