# Supplementary material for: Cellular Virotherapy Increases Tumor-Infiltrating Lymphocytes (TIL) and Decreases their PD-1+ Subsets in Mouse Immunocompetent Models
Source: Cancers (Basel). 2020 Jul 16;12(7):1920. doi: 10.3390/cancers12071920 (PMC7409201; doi:10.3390/cancers12071920)
Supplement: Supplementary file 1 [file cancers-12-01920-s001.pdf]

Supplementary Material: Cellular Virotherapy Increases Tumor-Infiltrating Lymphocytes (TIL) and Decreases their PD-1⁺ Subsets in Mouse Immunocompetent Models

Alvaro Morales-Molina, Miguel Ángel Rodríguez-Milla, Alicia Gimenez-Sanchez, Ana Judith Perisé-Barrios and Javier García-Castro

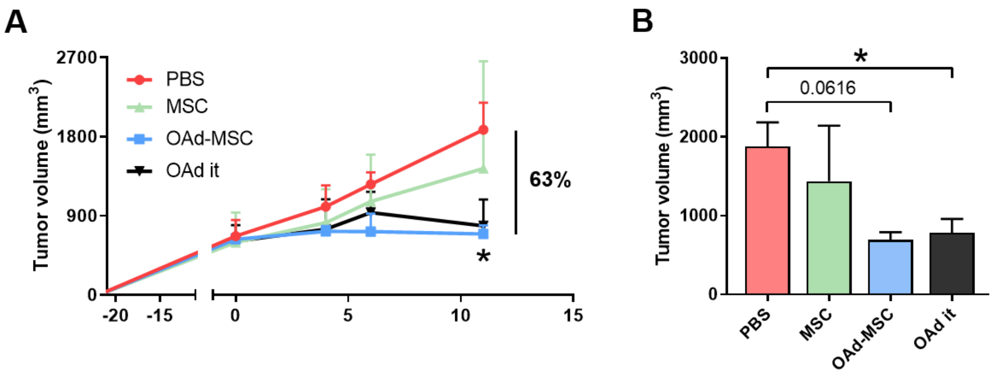

Figure S1. In vivo antitumor efficacy of dlE102 and OAd-MSCs in a mouse model of renal adenocarcinoma. (**A**) Follow-up of tumor volume in BALB/c mice bearing Renca tumors intraperitoneally treated with PBS (red), MSCs (green) or OAd-MSCs (blue), or intratumorally treated with dlE102 (OAd it, black). Data is represented as mean + SEM. (**B**) Tumor volume of control and treated groups at end point. Mann–Whitney U test, * $p < 0.05$.

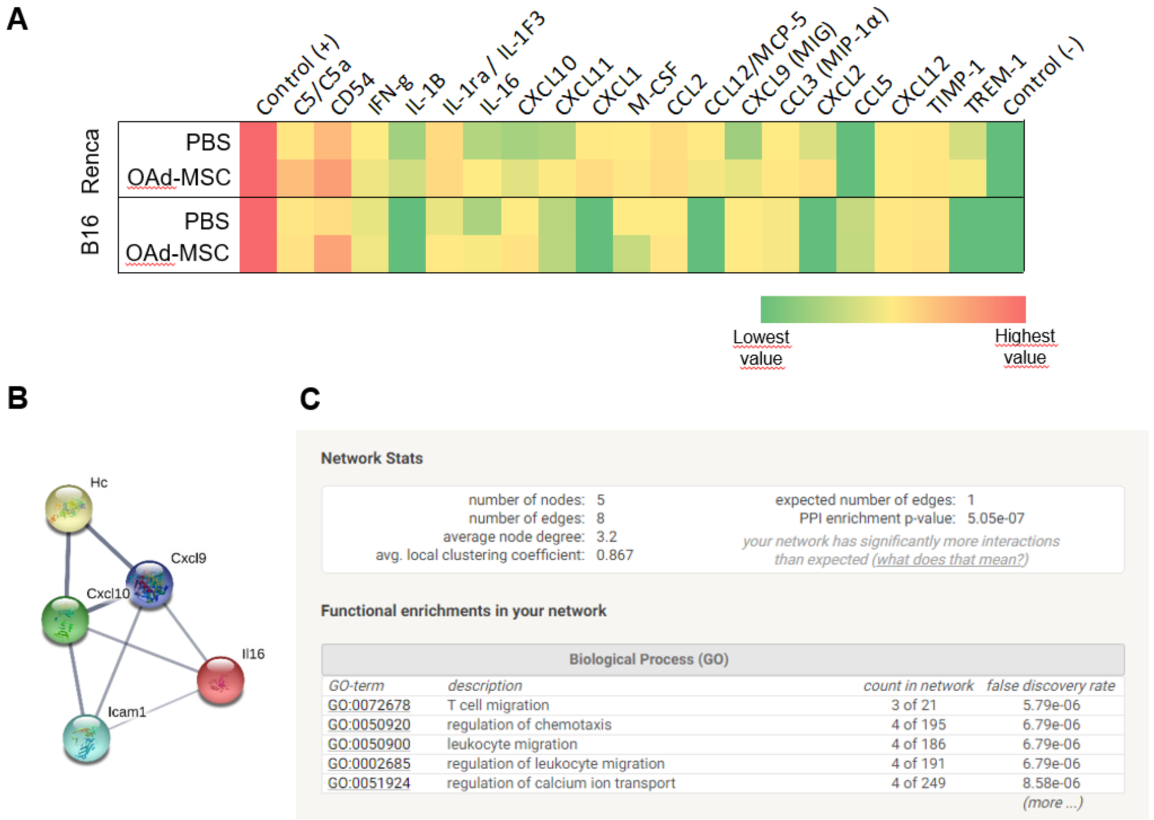

Figure S2. Pro-inflammatory profile in control and treated Renca and B16 tumors. (**A**) Heat-map of pro-inflammatory cytokines expressed in tumors treated with PBS or OAd-MSCs from models of renal adenocarcinoma (Renca) and melanoma (B16). Green indicates low expression, while red indicates high expression. (**B**) Network presenting cytokines with increased expression in both Renca and B16 tumors treated with OAd-MSCs. Line thickness indicates the strength of data support between edges. (**C**) Table shows results from the STRING analysis and the functional enrichments in the network according to the biological process.

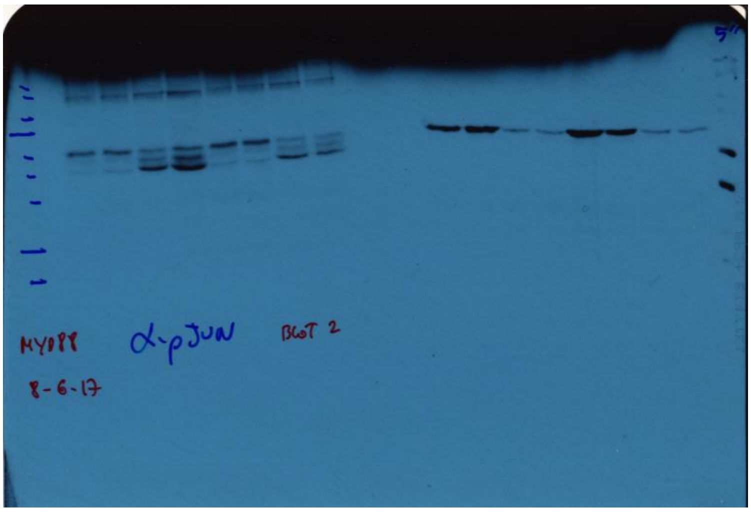

Figure S3. Complete WB of phospho-Jun (blot on the left), corresponding to Figure 1F. First four columns were cropped and included in the manuscript. From left to right: non-infected C57BL/6 MSCs at 3 h, MSCs infected with dlE102 (OAd-MSCs) at 3 h, non-infected MSCs at 24 h, MSCs infected with dlE102 (OAd-MSCs) at 24 h.

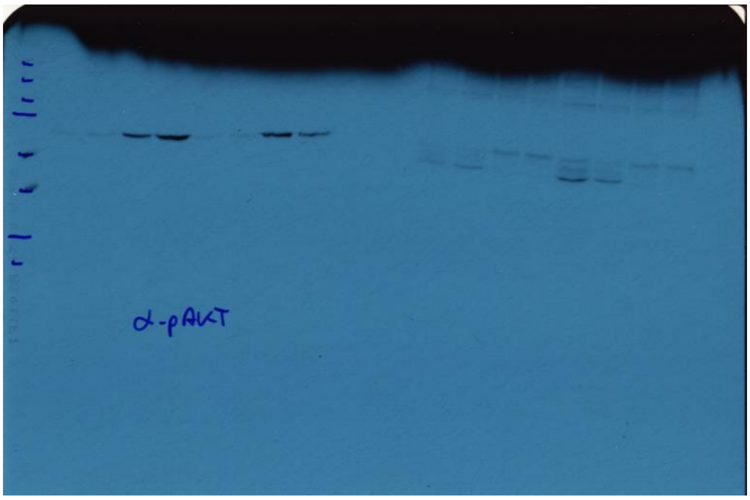

Figure S4. Complete WB of phospho-Akt (blot on the left), corresponding to Figure 1F. First four columns were cropped and included in the manuscript. From left to right: non-infected C57BL/6 MSCs at 3 h, MSCs infected with dlE102 (OAd-MSCs) at 3 h, non-infected MSCs at 24 h, MSCs infected with dlE102 (OAd-MSCs) at 24 h.

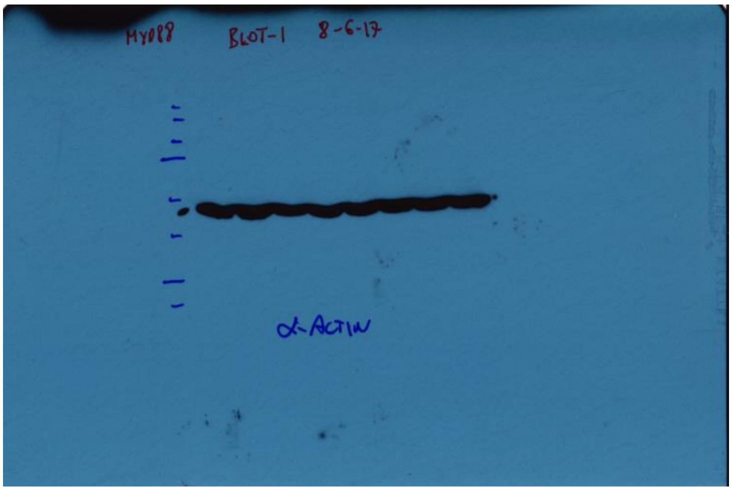

Figure S5. Complete WB of Actin, corresponding to Figure 1F. First four columns were cropped and included in the manuscript. From left to right: non-infected C57BL/6 MSCs at 3 h, MSCs infected with dlE102 (OAd-MSCs) at 3 h, non-infected MSCs at 24h, MSCs infected with dlE102 (OAd-MSCs) at 24 h.

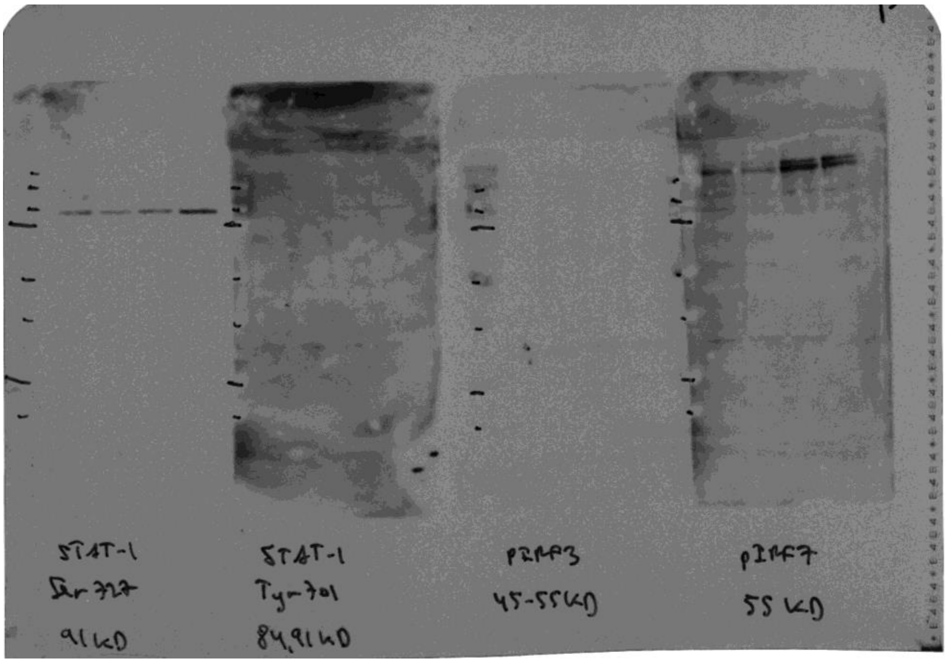

Figure S6. Complete WB of pospho-Stat-1 Ser727 (blot on the left), corresponding to Figure 1G. Images were cropped and included in the manuscript. From left to right: non-infected C57BL/6 MSCs at 3 h, MSCs infected with dlE102 (OAd-MSCs) at 3 h, non-infected MSCs at 24 h, MSCs infected with dlE102 (OAd-MSCs) at 24 h.

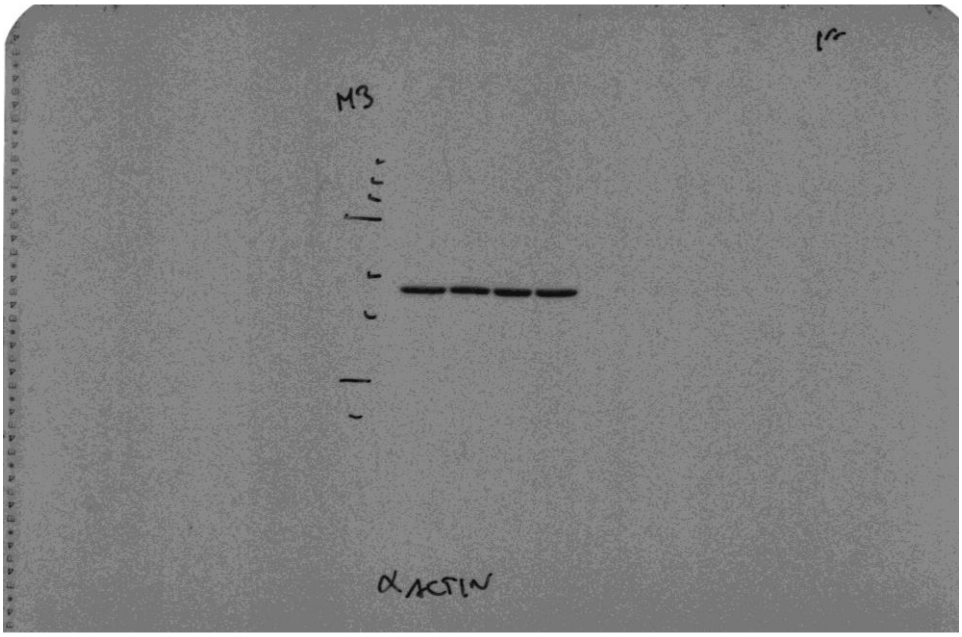

Figure S7. Complete WB of Actin, corresponding to Figure 1G. First four columns were cropped and included in the manuscript. From left to right: non-infected C57BL/6 MSCs at 3 h, MSCs infected with dlE102 (OAd-MSCs) at 3 h, non-infected MSCs at 24h, MSCs infected with dlE102 (OAd-MSCs) at 24 h.
